# Improving the fixed charge density of sustainably produced saloplastic anion exchange membranes†

**DOI:** 10.1039/d5su00221d

**Published:** 2025-06-17

**Authors:** Hestie A. Brink, Ricardo P. Martinho, Wiebe M. de Vos, Saskia Lindhoud

**Affiliations:** a Department of Membrane Science and Technology, University of Twente Enschede Netherlands; b Department of Molecules and Materials, University of Twente Enschede Netherlands s.lindhoud@utwente.nl

## Abstract

Recent studies have shown that sustainable ion exchange membranes can be fabricated by hot-pressing polyelectrolyte complexes (PECs), resulting in saloplastic membranes. Among these, the anion exchange membrane (AEM) formed from the strongly charged polyelectrolyte pair, poly(sodium 4-styrenesulfonate) (PSS) and poly(diallyl-dimethylammonium chloride) (PDADMAC) stands out due to its excellent chemical stability. However, the performance of this membrane is limited by its comparatively low fixed charge density. To address this limitation, we aimed to enhance the fixed charge density through incremental PDADMAC overcharging during the complexation step, followed by optimisation of hot-pressing conditions to produce dense, freestanding films. This approach allows for precise control over membrane charge and improves the reproducibility of films, thereby overcoming challenges in the processing and handling of non-stoichiometric PECs. NMR spectroscopy was used to quantify the fixed charge of the saloplastic AEMs before and after testing, providing a reliable and time-efficient method for assessing stability. Our results showed that a PDADMAC overcompensation of ∼30 mol% optimised the fixed charge density without compromising membrane stability. The enhanced membrane exhibited an 84% improvement in ionic conductivity (4.3 ± 0.3 mS cm^−1^ in 0.5 M KCl) compared to the original membrane. Notably, all membranes displayed excellent permselectivity (>90%) in 0.1 M KCl, and at higher electrolyte concentrations, a moderate improvement in permselectivity was observed with the increase in fixed charge density. Overall, this study presents a simple yet effective methodology for quantifying and optimising the fixed charge density of saloplastic membranes, resulting in significantly improved performance.

## Abstract 2

Enhancement of fixed charge density in PSS-PDADMAC saloplastic membranes *via* PDADMAC overcharging leads to improved ion conductivity and permselectivity.

## Graphical abstract

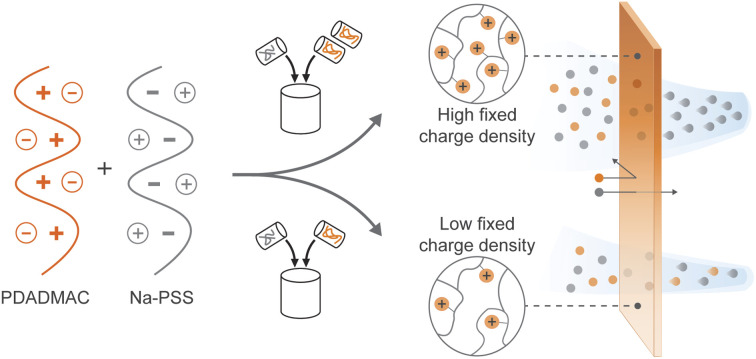

**Sustainability spotlight**

In the pursuit of sustainable materials and energy technologies, this study focuses on enhancing the performance of saloplastic ion exchange membranes (IEMs) by optimising their fixed charge density. These membranes are inherently sustainable due to their low toxicity, recyclability, and the use of saltwater as a solvent during fabrication, which aligns with SDG 12 (Responsible Consumption and Production) by utilising environmentally friendly materials and processes. Additionally, the use of the PSS-PDADMAC polyelectrolyte pair ensures excellent chemical stability, making these membranes suitable for long-term use in harsh environments. With improved performance and stability, these membranes are well-suited for advanced electrochemical technologies such as electrodialysis and fuel cells, contributing to SDG 6 (Clean Water and Sanitation) and SDG 7 (Affordable and Clean Energy). Furthermore, this work supports SDG 13 (Climate Action) by advancing sustainable energy technologies that reduce dependence on fossil fuels.

## 1. Introduction

Ion exchange membranes (IEMs) play a pivotal role in a wide range of electrochemical applications, including water treatment, electrochemical synthesis, energy storage, and energy conversion.^1,2^ These membranes are typically made from polymeric materials that contain a high concentration of fixed charged groups.^2,3^ In electrochemical cells, IEMs serve as selective barriers between the electrode compartments, permitting the passage of oppositely charged counterions, while retaining co-ions. This selective ion transport is crucial for the efficiency and lifespan of electrochemical cells, as it prevents the mixing of electrolyte solutions and directs ions to the appropriate compartments.^4–8^

When developing new IEMs, it is crucial to tailor the mechanical and chemical stability to suit specific applications,^7,9^ while also considering sustainability in material selection and manufacturing. The performance of IEMs is primarily evaluated based on their resistance and permselectivity, as these factors directly influence energy consumption and process efficiency.^1,4^ These properties are strongly influenced by the charge and hydration of the membrane, which can be quantified by a single parameter known as the fixed charge density, expressed as the number of fixed charges per litre of hydrated membrane.^1^

IEMs often face a concentration-induced trade-off between permselectivity and conductivity.^1,4,10^ While higher ion concentrations enhance conductivity due to the increased availability of mobile ions, it is typically accompanied by a reduction in permselectivity due to a phenomenon known as charge screening.^10^ The high number of ions near the membrane surface reduces the electrostatic interactions between the fixed charged groups and the ions in solution, allowing undesired ions to pass more easily through the membrane. To mitigate this trade-off, the fixed charged density of the IEM should be improved,^1,10^ either by incorporating more charged groups into the membrane or by reducing water uptake, as water dilutes the charges. A higher fixed charge density enhances the Donnan potential,^1,10,11^ thereby improving permselectivity by the preferential transport of counterions over co-ions. Additionally, this increase in fixed charge density also improves conductivity by providing more charge carriers.^1,10,11^

**Fig. 1:**
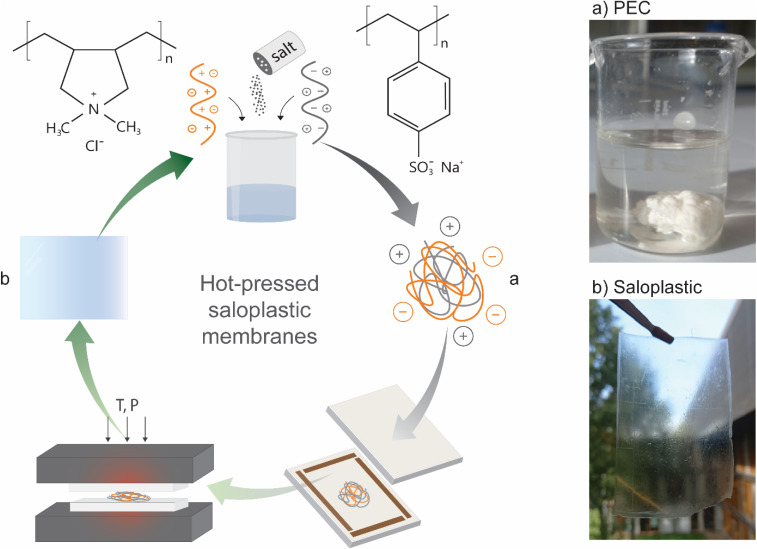
Scheme of hot-pressing polyelectrolyte complexes (a) into saloplastic ion-exchange membranes (b).

A novel method for fabricating IEMs has recently been developed through the hot-pressing of non-stoichiometric polyelectrolyte complexes (PECs).^12^ PECs form when oppositely charged polyelectrolytes are mixed together, creating ionic crosslinks that result in a solid precipitate.^13^ These complexes are processed in the presence of salt, which dopes the PEC by breaking ionic crosslinks, allowing the material to soften and be moulded.^14,15^ Because salt plays a key role in plasticising these materials, the resulting products are commonly referred to as “saloplastics”.^14–16^ Therefore, saloplastic IEMs can be fabricated using only saltwater, eliminating the need for toxic solvents commonly used in membrane production. Furthermore, saloplastic materials have a high recyclability potential^17–19^ which further highlights the sustainability of this approach. A scheme of this hot-pressing process is given in Fig. 1. Of particular interest is an anion exchange membrane produced from the strongly charged polyelectrolyte pair, poly(diallyl-dimethylammonium chloride) (PDADMAC) and poly(sodium 4-styrenesulfonate) (PSS),^20^ which exhibits remarkable pH stability (pH 1–14). However, while this membrane is mechanically stable and monovalent ion selective, its conductivity and permselectivity are comparatively low due to its low fixed charge density.^20^

Previous studies have shown that high levels of PDADMAC overcompensation can be achieved in PSS-PDADMAC PECs, with clear correlations between molar mixing ratios and PEC composition.^21,22^ This suggests that the fixed charge density of the saloplastic anion exchange membrane (AEM) could be increased by overcharging with PDADMAC. However, a follow-up study aimed at controlling the fixed charge density of these membranes by hot-pressing PECs with varying molar ratios yielded unexpected results.^23^ It found that overcharging with PDADMAC (1 : 2 and 1 : 3) did not improve the charge or performance of the AEM. Instead, the 1 : 1 molar ratio was the most effective for the PSS-PDADMAC system, as membranes produced from non-stoichiometric mixing ratios were difficult to process and handle.^23^

This discrepancy may stem from differences in the complexation conditions used. The study on saloplastic IEMs investigated quite extreme overcompensation levels,^23^ while successful studies typically used more moderate ratios.^21,22^ This is consistent with the fact that complexation is a kinetically limited process,^17^ strongly influenced by factors such as polyelectrolyte molar ratio, polyelectrolyte concentration, salt concentration, and the order of mixing.^16^ Additionally, the method used to quantify the charge of the saloplastic membranes may not have been sensitive enough to detect small charge differences, as indicated by the relatively large error margins reported.^23^

To optimise the fixed charge density of saloplastic IEMs, it is crucial to accurately quantify their charge. The charge of IEMs is assessed by measuring the ion exchange capacity, which generally employs titration methods to measure the number of ions exchanged per unit weight of membrane.^24,25^ However, saloplastic membranes present a unique challenge, as they contain both fixed anionic and cationic groups, necessitating the measurement of both anion and cation exchange capacities.^20^ This dual measurement can increase potential errors, particularly when high salt concentrations (≥1 M) are utilised for rapid ion exchange. Under these conditions, the structure and stability of the saloplastic membrane may be compromised by the effects of salt doping.^14^ Consequently, there is an urgent need for enhanced methods to accurately quantify the fixed charge of saloplastic membranes.

This study builds on our previous research by enhancing the fixed charge density of PSS-PDADMAC saloplastic AEMs through controlled overcharging of PDADMAC. We employ a systematic approach that involves incrementally adjusting the PDADMAC content during the complexation step, followed by the optimisation of hot-pressing conditions to mitigate swelling and viscoelastic changes resulting from variations in PEC stoichiometry. To accurately quantify the membrane's composition and charge, we employ ^1^H-NMR, overcoming the limitations of traditional ion exchange capacity tests. Membrane performance is then evaluated by measuring permselectivity and conductivity at varying ion concentrations to assess the trade-off between these properties. This work presents an improved methodology for fabricating non-stoichiometric saloplastic IEMs, resulting in a significant enhancement of fixed charge density and overall membrane performance.

## 2. Materials and methods

### 2.1. Materials

Poly(diallyldimethylammonium chloride) (PDADMAC, average molecular weight = 400–500 kg mol^−1^), poly(sodium 4-styrenesulfonate) (PSS, average molecular weight = 1000 kg mol^−1^), potassium bromide (KBr, ACS reagent grade ≥99.0%), potassium chloride (KCl, ACS reagent grade, 99.0–100.5%) and deuterium oxide (D_2_O, 99.9 atom % D) were purchased from Sigma-Aldrich. Due to variability in solids content between polyelectrolyte batches, the actual wt% of PSS and PDADMAC were verified gravimetrically with oven drying and used in subsequent calculations. MilliQ water from a Millipore Synergy^®^ Water Purification System was used for dilutions.

### 2.2. Polyelectrolyte complexation

PECs were prepared with 0, 10, 20, 30 and 40 mol% excess PDADMAC based on the number of monomer repeat units. The polyanion and polycation solutions were prepared separately and then mixed together in a third beaker for 15 minutes with stirring.^17^ The polyanion and polycation solutions contained 250 mM KBr and the polyelectrolyte concentrations were adjusted according to the desired molar ratio.^12,17^ The combined polyelectrolyte concentration was kept constant at 25 g L^−1^ during complexation and 10 g of PEC was prepared for each molar ratio.

The PEC precipitate was washed, cut into smaller pieces and soaked in MilliQ water for 24 hours to remove excess KBr. The MilliQ water was refreshed several times to ensure removal of excess salt from the PEC. The PEC was then oven dried at 105 °C, ground to a powder and stored in an airtight container.^22^ This was done to ensure that the PEC weight, moisture and salt content can be controlled during the membrane preparation process.

For each membrane (5.5 × 8.5 cm^2^), 1.7 g of PEC powder was carefully weighed and soaked in 100 mL of the desired KBr solution (0.2–0.3 M) for at least 24 hours prior to hot-pressing. Due to changes in the viscoelastic properties of non-stoichiometric PECs, it was necessary to adjust the hot-pressing salt concentration in order to regulate the elasticity of the films. Consequently, highly overcompensated PECs were processed at lower salt concentrations (0.2 M KBr). A summary of the complexation conditions are given in Table 1.

**Table 1:** A summary of the complexation and hot-pressing conditions used to prepare saloplastic membranes

	mol% excess PDADMAC in mixing solutions				
	0	10	20	30	40
**Polyanion solution**					
Volume (mL)	200	200	200	200	200
KBr concentration (mM)	250	250	250	250	250
PSS concentration^a^ (mM)	**136**	**130**	**125**	**120**	**116**

**Polycation solution**					
Volume (mL)	200	200	200	200	200
KBr concentration (mM)	250	250	250	250	250
PDADMAC concentration^a^ (mM)	**136**	**143**	**150**	**156**	**162**

**Membrane preparation**					
PEC	Soak 1.7 g dry PEC powder in KBr solution for 24 hours	Soak 1.7 g dry PEC powder in KBr solution for 24 hours	Soak 1.7 g dry PEC powder in KBr solution for 24 hours	Soak 1.7 g dry PEC powder in KBr solution for 24 hours	Soak 1.7 g dry PEC powder in KBr solution for 24 hours
KBr concentration (mM)	**300**	**300**	**300**	**200**	**200**
PEC moisture content (wt%)	∼55	∼55	∼55	∼55	∼55
Plasticisation	45 min at 90 °C	45 min at 90 °C	45 min at 90 °C	45 min at 90 °C	45 min at 90 °C
Pressing	10 min at 90 °C and 150 bar	10 min at 90 °C and 150 bar	10 min at 90 °C and 150 bar	10 min at 90 °C and 150 bar	10 min at 90 °C and 150 bar
Cooling	∼90 min until temperature <30 °C	∼90 min until temperature <30 °C	∼90 min until temperature <30 °C	∼90 min until temperature <30 °C	∼90 min until temperature <30 °C

a Polyelectrolyte concentrations based on number of monomer repeat units.

### 2.3. Membrane preparation

Hot-pressed moulds were made of rectangular Delrin endplates (15 × 8 × 0.5 cm^3^) with a 0.122 mm PTFE coated fiberglass spacer (Lubriglas-CHAP-1540, Reichelt Chemietechnik GmbH + Co, Heidelberg, Germany) glued to the bottom endplate.^20^ Four thin outlets were cut into the corners of the 5.5 × 8.5 cm^2^ spacer to allow excess PEC and moisture to escape. The KBr soaked PEC (∼55 wt% moisture) was placed in the middle of the spacer of the bottom endplate and covered with the top endplate.

The mould was then placed in a pre-heated (90 °C) FV20R Rollie Driptech Rosin Press (purchased from FVR, Canada) and the PEC was allowed to plasticise for 40 minutes, with the top plate touching the mould (0 bar). After the plasticisation time, the PEC should be soft and transparent. The endplates of the hot-press was then slowly closed until a final pressure of 150 bar was applied to the mould. After 20 minutes, the temperature setpoint was lowered to 25 °C and the hot-pressed complex was allowed to cool under pressure until the hot-press temperature dropped below 30 °C. The thin transparent film was then removed from the mould and stored in an airtight bag. A summary of the hot-pressing conditions are given in Table 1.

A minimum of three membranes were prepared from each PEC. The membranes were cut into smaller samples (diameter of 2 cm) and equilibrated in the electrolyte solution of interest for at least 24 hours prior to testing.

### 2.4. Saloplastic composition

The molar ratios of the PSS-PDADMAC saloplastic membranes were quantified using proton nuclear magnetic resonance spectroscopy (^1^H-NMR). All NMR measurements were conducted using a Bruker 14.1 T magnet (600.16 MHz for ^1^H) operated by an Avance Neo console operating a two-channel broadband probe (BBO), with temperature set to 25 °C. Samples were prepared by dissolving oven dried saloplastic membranes in 2.5 M KBr in D_2_O at a concentration of 20 mg mL^−1^. Ratiometric analysis of the composition of the complexes was performed as described in ESI (eqn (S1)),† based on previously described methods.^17,26,27^

### 2.5. Water uptake

The water uptake of the saloplastic membranes was measured gravimetrically after equilibrating saloplastic samples (∼2 cm^2^) in the electrolyte solutions at 25 °C for 24 hours. The equilibrated samples were then removed from the solution, wiped with tissue paper to remove surface water and weighed. The samples were dried in an oven at 105 °C and the dry weight was recorded. The water uptake was reported as the mean of five measurements, with the 95% confidence interval indicated as the error margin.
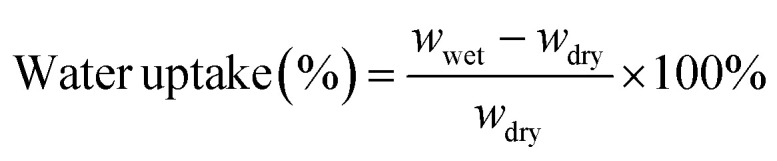
where *w*_wet_ is the wet weight of the equilibrated membrane and *w*_dry_ is the dry weight of the membrane.

### 2.6. Mechanical strength

The Young's modulus is defined as the ratio of tensile stress to tensile strain in the linear elastic region of a material and provides valuable information about the stiffness and elasticity of polymeric materials.^28,29^ A high Young's modulus indicates that the material is more rigid and brittle, whereas a low Young's modulus suggests that the material is more flexible and capable of undergoing greater deformation.^30^

The mechanical strength of the saloplastic films was measured using a electromechanical testing system (Instron 5800) at 25 °C and a speed of 2 mm min^−1^. Samples were cut into 5 × 50 mm^2^ strips, according to the ASTM D882 standard for thin (<1 mm) polymer films.^31^ Measurements were performed in triplicate.

### 2.7. Permselectivity

Permselectivity describes how well IEMs distinguish between anions and cations, *i.e.* how selective membranes are towards counterions over co-ions.^32^ Permselectivity is calculated as the ratio of the measured chemical potential difference (Δ*φ*_measured_) to the theoretical potential (Δ*φ*_Nernst_) for a 100% selective membrane when a ionic concentration gradient is applied across the membrane.^33^

A ionic concentration gradient was established by positioning the IEM between two chambers circulating different concentrations of the same electrolyte solution (KCl).^32^ The potential difference across the membrane was then measured using calomel reference electrodes (VWR, The Netherlands) positioned near the membrane in each chamber. The concentration of salt in the high concentration chamber was always five times greater than that of the low concentration chamber. The electrolyte solutions were circulated through a thermostatic bath to control the temperature at 25 °C. The permselectivity was reported as the average of a minimum of five measurements, with the 95% confidence interval indicated as the error margin.
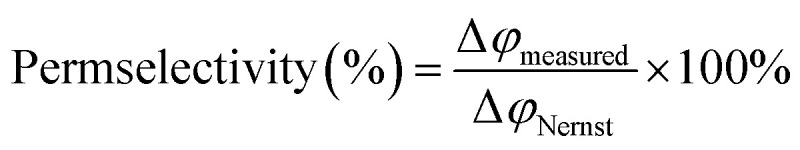

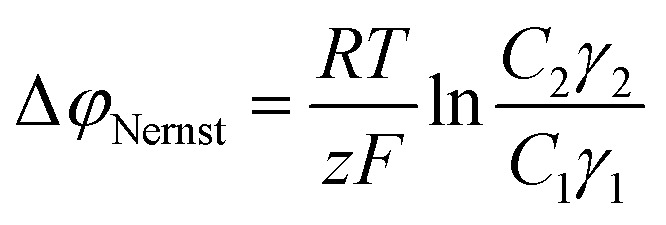
where Δ*φ*_measured_ is the measured chemical potential difference (mV), Δ*φ*_Nernst_ is the theoretical potential (mV), *R* is the gas constant (J mol^−1^ K^−1^), *T* the temperature (K), *z* the electrochemical valence, *F* the Faraday constant (96 485C mol^−1^), *C*_1_ and *C*_2_ the concentrations of the electrolyte solutions (M) and *γ*_1_ and *γ*_2_ the activity coefficients of the electrolyte solutions.

### 2.8. Ionic conductivity

Ionic conductivity describes how well IEMs conduct ions, and is expressed as the reciprocal of the membrane electrical resistance normalised for membrane thickness.^2^ As this study did not focus on optimising membrane thickness, it was considered appropriate to report ion conductivity, which is a material property, rather than electrical resistance, which is related to membrane thickness.

A six-compartment cell (Fig. S1†) made from Plexiglas was used for electrical resistance measurements.^34,35^ The test membrane was inserted between two disks with an open area of 1.77 cm^2^ and secured between the two middle compartments (3 and 4). A commercially available cation exchange membrane (Neosepta CMX from Astom Corporation, Japan) was inserted between the other compartments to separate the electrolyte solutions. The middle compartments (3 and 4) were circulated with the test electrolyte solution (0.1, 0.3 or 0.5 M KCl), while the adjacent compartments (2 and 5) were circulated with 0.5 M KCl. The ends of the outer compartments were fitted with platinum coated titanium electrodes to apply current to the cell. To prevent chlorine gas from forming, the outer compartments (1 and 6) were circulated with 0.5 M K_2_SO_4_. Haber–Luggin capillaries were positioned close to the test membrane in the two central compartments and were connected to calomel reference electrodes (VWR, The Netherlands) to measure the voltage drop across the test membrane.

The DC resistance was calculated as the slope of the IV curve which was generated by applying stepwise currents ranging from 0 to 15 mA to the platinum electrodes and measuring the corresponding voltage drop across the membrane using a Metrohm Autolab potentiostat (PGSTAT302N, The Netherlands). The membrane resistance was calculated by subtracting the solution resistance,^34,35^ which was quantified by testing the response without the test membrane. The ionic conductivity was reported as the average of a minimum of five measurements,^2^ with the 95% confidence interval indicated as the error margin.
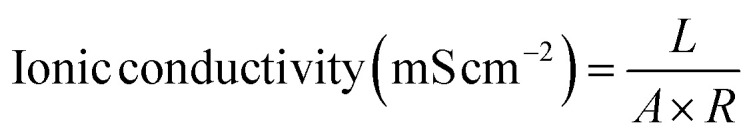
where *L* is the membrane thickness measured using a micrometer, *R* is the membrane resistance (Ω) and *A* is the membrane area (1.77 cm^−2^).

## 3. Results and discussion

### 3.1. Preparation and charge control of saloplastic AEMs

The aim of this study was to enhance the fixed charge density of PSS-PDADMAC saloplastic AEMs by overcharging with PDADMAC. This was achieved by systematically adjusting the polyelectrolyte molar ratio during the complexation step to introduce varying levels of PDADMAC overcompensation. We observed that the behaviour of the complexes during hot-pressing was strongly influenced by the PEC stoichiometry. In particular, complexes with a higher PDADMAC content were often too thin and sticky to remove from the mould, which we attributed to a decrease in viscosity during the plasticisation step. To address this issue, we refined our hot-pressing method to better control moisture content and salt concentration throughout the process. Instead of directly hot-pressing the wet complex,^12,20^ we first dried and then rehydrated the PEC in the required salt solution (Table 1), allowing for improved control over viscosity. Through this modification, we determined that PECs with higher PDADMAC content should be soaked in a lower salt concentration (200 mM instead of 300 mM KBr) to reduce stickiness and improve uniformity. As a result, we not only resolved reproducibility issues, but also overcame previously reported processing and handling challenges^23^ associated with saloplastic membranes made from non-stoichiometric molar ratios.

Another challenge was quantifying the charge in saloplastic membranes. As discussed earlier, saloplastic membranes contain both anionic and cationic groups,^20^ which makes conventional methods based on measuring exchanged counterions less accurate and more time-consuming. To address this, we utilised the ^1^H-NMR method, originally developed for determining the molar ratio of extruded PSS-PDADMAC PECs, to quantify the polyelectrolyte molar ratio within the saloplastic films (eqn (S1) and (S2)†).^17^ The NMR analysis enabled us to quantify the molar excess of PDADMAC, which was then used to calculate the ion exchange capacity of the membranes (eqn (S3)†). This approach offers a new and effective means of quantifying the net charge in saloplastic membranes.

**Fig. 2:**
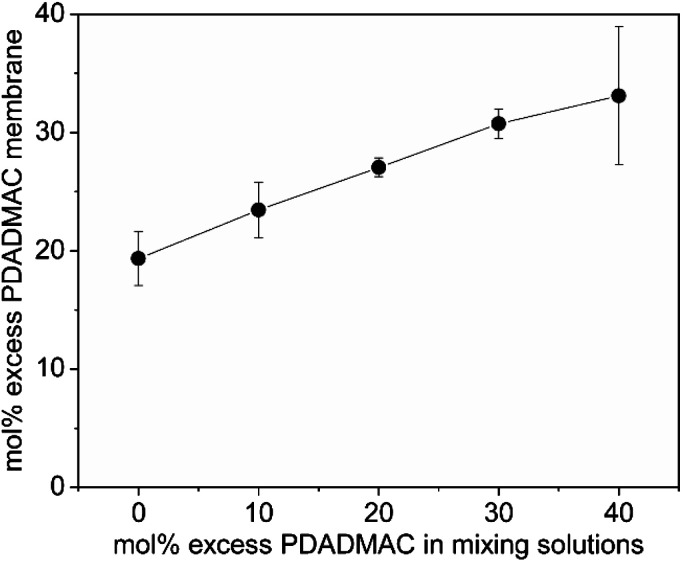
Relationship between the molar excess of PDADMAC used in the mixing solution during complexation and the molar excess of PDADMAC incorporated into the resulting membrane. Each point represents the mean of at least three measurements, with the 95% confidence interval indicated as the error margin. Lines are guides for the eye to highlight the general trend of the data.

Fig. 2 shows the composition (mol% excess PDADMAC) of pristine saloplastic membranes that were hot-pressed from PECs prepared at varying stoichiometries (based on monomer repeat units). It is evident that PEC complexation did not align with the initial mixing ratios. Instead, the complexation process favoured PDADMAC, as evidenced by the formation of a membrane containing 19 ± 2 mol% excess of PDADMAC when equimolar solutions were combined. This is most likely due to a higher uptake of PDADMAC in the PEC, with a larger proportion of PSS remaining in the supernatant. Another possible explanation could be the presence of impurities in the polyelectrolytes; however, ^1^H-NMR analysis did not reveal any impurities. Notably, increasing the PDADMAC content in the mixing solutions led to a proportional increase in the PEC PDADMAC content. This suggests that our approach provides significant control over the charge of the resulting membranes, which is particularly noteworthy given the inherent challenges associated with controlling this kinetically driven complexation step.^17^

These findings are consistent with other studies that showed that the PSS-PDADMAC system often overcompensates with PDADMAC under similar complexation conditions.^20,21,23^ PDADMAC overcompensation is a phenomenon that also occurs when producing polyelectrolyte multilayer membranes, including PSS-terminated ones. These studies report that PDADMAC overcompensation is more prominent at higher ionic strengths and has been attributed to the higher mobility of PDADMAC in the complex compared to PSS.^36–38^

As shown in Fig. 2, a maximum overcompensation of 33 ± 5 mol% was achieved with a 40 mol% excess of PDADMAC in the mixing solutions, demonstrating the successful enhancement of the charge of the saloplastic AEMs. Notably, this data point also shows a relatively large error, which will be addressed in the following section, where we investigate the swelling behaviour, quantify fixed charge density and discuss PEC stability.

### 3.2. Water uptake and fixed charge density

Water uptake is an important parameter to consider as it strongly influences the performance of IEMs. An increase in swelling reduces the fixed charge density, as water molecules dilute the charges within the membrane. This dilution lowers the effect of Donnan exclusion,^1,11^ thereby allowing more co-ions to pass and consequently reducing the selectivity of the membrane.

**Fig. 3:**
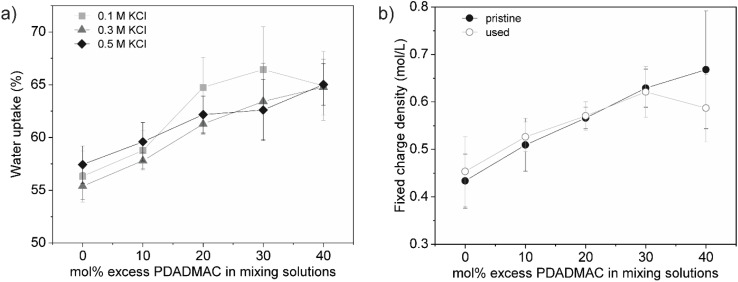
(a) Water uptake of saloplastic membranes at different KCl concentrations, measured at 25 °C. (b) Fixed charge density of saloplastic membranes in both pristine form and after testing and long-term storage in 0.5 M KCl (used). Each point represents the mean of at least three measurements, with the 95% confidence interval indicated as the error margin. Lines are guides for the eye to highlight the general trend of the data.

Fig. 3a shows the water uptake of the membranes at varying salt concentrations, which correspond to the electrolyte solutions used in subsequent membrane performance tests. While no clear relationship between electrolyte concentration and water uptake was observed, water uptake increased with higher levels of PDADMAC overcompensation. Membranes with higher concentrations of fixed charges require more counterions to maintain electroneutrality. The presence of these counterions, along with their associated hydration shells, contributes to the observed increase in water uptake.^11,39^

The water uptake results, along with the membranes' composition (Fig. 2), were then used to calculate the fixed charge density (eqn (S4)†). To evaluate the stability of the PECs, the membrane composition (mol% excess PDADMAC) was re-analysed using NMR after testing and storage in the electrolyte solution with the highest concentration (0.5 M KCl) for at least three months. The fixed charge density of the used membranes was then compared to that of the pristine membranes.

As shown in Fig. 3b, a steady increase in fixed charge density was observed with higher levels of PDADMAC overcompensation in the pristine membranes. The first four saloplastic membranes exhibited excellent stability, with no significant changes in composition after testing and storage. In contrast, the membrane prepared with the highest amount of PDADMAC showed a reduction in PDADMAC overcompensation, resulting in a loss of charge relative to the pristine membrane. Initially, this membrane contained 33 ± 5 mol% excess PDADMAC, which aligns with the maximum level of PDADMAC overcompensation reported in previous studies.^22,40^ This suggests that the PEC has reached a point of instability, resulting in the leaching of PDADMAC over time, which is expected to negatively impact membrane performance.

It is expected that the increased membrane charge, along with the higher water uptake, will also influence the viscoelastic properties of the saloplastic membranes, given that water acts as a plasticiser for these materials.^17^ To explore the broader implications of water uptake, we next examine its effect on the mechanical properties of the membranes.

### 3.3. Mechanical properties

Mechanical stability is essential for IEMs to maintain durability and reliable performance under varying operational conditions.^28–30^ A membrane that is too stiff may crack under stress, while one that is too flexible could lose its structural integrity, impacting its selectivity and efficiency. The Young's modulus is a useful measure of mechanical stability, with a high value indicating rigidity and brittleness, and a low value suggesting flexibility and the ability to deform.^30^ We measured the Young's modulus in both dry and hydrated states to assess how the elasticity of our saloplastic IEMs changes with increased charge and hydration.

**Fig. 4:**
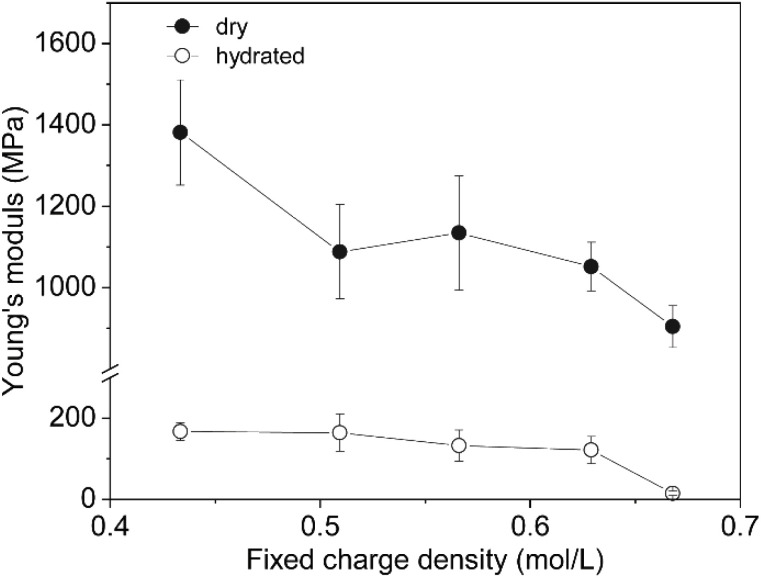
Young's modulus of dry and hydrated (equilibrated in 0.5 M KCl) saloplastic membranes at room temperature. Each point presents the average of at least three measurements with the 95% confidence interval indicated as the error margin. Lines are guides for the eye to highlight the general trend of the data.

As shown in Fig. 4 the Young's modulus of the saloplastic membranes was significantly lower in the hydrated state compared to the dry state. This difference can be attributed to the plasticising effect saltwater has on saloplastics, which makes the membranes softer and more flexible.^17^ Furthermore, a reduction in Young's modulus was observed at higher levels of PDADMAC overcompensation, even in the dry state. This decrease is likely due to the excess PDADMAC introducing additional extrinsic sites, which reduce the crosslink density and thus the material's mechanical strength.^14^ It is also possible that the observed reduction in Young's modulus is partially due to increased water uptake from the air, as the membranes with higher PDADMAC content may be more hydrophilic.^22^

These results are consistent with the findings of Chen *et al.*,^22^ who observed lower moduli and glass transition temperatures in non-stoichiometric PSS-PDADMAC complexes. Specifically, the glass transition temperature decreased from 35 °C for the stoichiometric complex to approximately 20 °C for PECs with a PDADMAC molar excess greater than 20%.^22^ In a separate study, this group also reported similar reductions in moduli and glass transition temperatures with increased salt concentration.^41^

In summary, overcharging with PDADMAC clearly increases the elasticity of the membrane. Other studies have also shown that factors such as temperature and salt concentration affect their mechanical stability. Based on the low Young's modulus of the highly overcompensated membranes, their use in applications requiring higher salt concentrations and temperatures is likely limited.

### 3.4. Membrane performance

The performance of IEMs is largely governed by their ionic conductivity and permselectivity. While high conductivity facilitates ion transport, high permselectivity ensures effective ion separation.^32^ However, a trade-off often exists between these two properties, where increased electrolyte concentration improves conductivity but reduces permselectivity.^1,4,10^ In this study, we successfully increased the fixed charge density of our saloplastic AEMs through PDADMAC overcharging. To evaluate whether this resulted in an overall improvement in membrane performance, we measured conductivity and permselectivity at various electrolyte concentrations and examined their relationship to the fixed charge density.

**Fig. 5:**
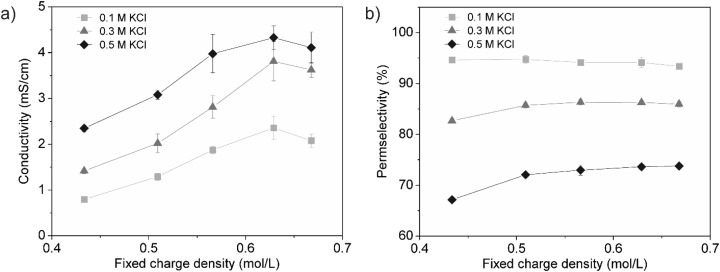
Effect of fixed charge density on (a) conductivity and (b) permselectivity of saloplastic AEMs in different electrolyte concentrations at 25 °C. Each point represents the average of a minimum of five measurements, with the 95% confidence interval indicated as the error margin. If no error bar is visible, the error is smaller than the marker. Lines are guides for the eye to highlight the general trend of the data.

As shown in Fig. 5a, conductivity increased with both electrolyte concentration and fixed charge density, but plateaued at the highest fixed charge density. This plateau can be attributed to the leaching of PDADMAC from the PEC over time, leading to a loss of charge, as was shown in Fig. 3b. Increasing the fixed charge density in IEMs provides more charged sites, which enhances ion transport within the membrane.^1,11^ Simultaneously, a higher electrolyte concentration increases the number of available ions in the solution, which improves ion exchange and further enhances conductivity.^1,10^ A maximum conductivity of 4.3 ± 0.3 mS cm^−1^ was achieved at a fixed charge density of 0.63 ± 0.4 mol L^−1^ in 0.5 M KCl, corresponding to a resistance of 4.7 Ω cm^2^ for a 200 μm membrane. This represents an impressive 84% improvement compared to the original membrane (prepared from the 1 : 1 molar ratio).^20,23^ Since resistance is influenced by membrane thickness, further improvement could be achieved by fabricating thinner membranes in future studies.

Fig. 5b shows that all membranes exhibit excellent permselectivity at 0.1 M KCl; however, permselectivity decreases as the electrolyte concentration increase. This trend highlights the inverse relationship between permselectivity and conductivity, emphasizing the concentration induced trade-off between these properties.^1,4,10^ The reduction in permselectivity can be attributed to charge screening, which weakens Donnan exclusion and reduces the membrane's ability to discriminate between ions.^10^

At higher electrolyte concentrations (0.3 and 0.5 M KCl), a slight improvement in permselectivity was observed with the initial increase in fixed charge density, but it remained relatively constant thereafter. This was unexpected, as increasing the fixed charge density of IEMs typically enhances permselectivity by strengthening the Donnan potential, which more effectively excludes co-ions.^11^ However, the observed trend can be attributed to the loss of mechanical stability in the highly charged membranes. During permselectivity testing, the membranes with higher charge densities stretched and turned white (Fig. S3†), particularly at higher electrolyte concentrations. This indicates increased swelling and porosity,^42–44^ which typically reduces permselectivity. This observation aligns with the Young's modulus results presented in Fig. 4, which showed that increasing the fixed charge density increased the elasticity of the saloplastic membranes, leading to the loss of structural integrity during the permselectivity tests.

In summary, increasing the fixed charge density resulted in a significant improvement in conductivity and a modest enhancement in permselectivity. The levelling off of permselectivity can be attributed to a loss of mechanical stability, a challenge that can be easily overcome by reinforcing the membranes with mesh or fabric, a common approach in commercial membranes.^30^ The feasibility of reinforcing saloplastic films with woven and non-woven porous substrates has already been demonstrated in previous work, where such reinforcement led to an increase in Young's modulus.^12^ In addition to mechanical reinforcement, future work could also explore the use of natural polymers to improve the sustainability of saloplastic IEMs. Natural polyelectrolytes such as chitosan, alginate, and cellulose derivatives,^45–47^ have been successfully used in membrane applications and could offer a more environmentally friendly alternative to synthetic polymers.

## 4. Conclusion

This study successfully demonstrates the enhancement of fixed charge density in PSS-PDADMAC saloplastic AEMs through controlled overcharging with PDADMAC, resulting in significant improvements in membrane performance. The systematic approach used for varying PDADMAC content and optimising hot-pressing conditions has proven effective in increasing the fixed charge density while overcoming previous challenges in membrane fabrication and handling.

A key innovation of this work is the use of ^1^H-NMR spectroscopy to quantify the fixed charge density in saloplastic AEMs. This technique overcomes the limitations of traditional titration methods, providing an accurate and time-efficient means of assessing the charge in saloplastic AEMs, which contain both anionic and cationic charge groups. Additionally, NMR can be employed to monitor the stability of the membrane after testing, providing valuable insights into how the membrane's composition and charge changes over time. With this, we successfully identified the optimal point for PDADMAC overcompensation (∼30 mol%), maximising fixed charge density without compromising membrane stability.

In this study, the ionic conductivity of PSS-PDADMAC AEMs was improved by a remarkable 84%, marking a significant step forward in the development of sustainable, high-performance ion exchange membranes. A moderate improvement in permselectivity was also observed; however, this was limited by increased water uptake and the loss of mechanical stability due to the higher fixed charge density. Future work will focus on reinforcing the membranes to improve their mechanical stability, reduce swelling, and enhance permselectivity. With enhanced mechanical stability, subsequent studies will investigate the effects of temperature, long-term operation, and chemical stability to better tailor the membranes for specific applications.

Overall, this research offers valuable insights into the development and optimisation of saloplastic IEMs. While this study focused on PSS-PDADMAC, the same approach could be extended to other polyelectrolyte systems, including those based on natural polymers. Exploring such systems in future work could further improve the sustainability of saloplastic IEMs.

## Conflicts of interest

There are no conflicts of interest to declare.

## Supplementary Material

SU-003-D5SU00221D-s001

## Data Availability

After publication data for this article, will become available available at [4TU.ResearchData] at [URL – format DOI https://doi.org/10.4121/940b5400-1b62-4929-93f9-75dbaef4d8b3].
