# Supplementary material for: Improving the fixed charge density of sustainably produced saloplastic anion exchange membranes
Source: RSC Sustain. 2025 Jun 17;3(8):3473–82. doi: 10.1039/d5su00221d (PMC12183635; doi:10.1039/d5su00221d)
Supplement: SU-003-D5SU00221D-s001 [file SU-003-D5SU00221D-s001.zip › supporting information_resubmission.pdf]

Supporting Information

1. Quantification of excess PDADMAC in saloplastic membranes

Quantification of the proportion of PDADMAC and PSS present in the saloplastic membranes was achieved from NMR spectra as the one demonstrated below (**Figure S2**). The peaks taken to represent each species were for PSS the whole aromatic region between 6.1 and 8.1 ppm, and for PDADMAC the peak corresponding to the ring CH between 2.4 and 3.1 ppm (based on prior assignments).[1, 2] The determination of the excess of PDADMAC was inspired by the work of Shamoun *et al.*[3] and done as:

$$\frac{[PDADMAC]}{[PSS]} = \frac{2I_{PDADMAC}}{I_{PSS}} \quad \text{Eq. S1}$$

$$mol\%\ excess\ PDADMAC = \left(\frac{[PDADMAC]}{[PSS]} - 1\right) \times 100 \quad \text{Eq. S2}$$

in which *I* correspond to the integrals of each species in the mentioned region. No significant difference in chemical shifts was observed throughout the samples, likely due to similar sample concentrations and the natural broadness of the peaks.

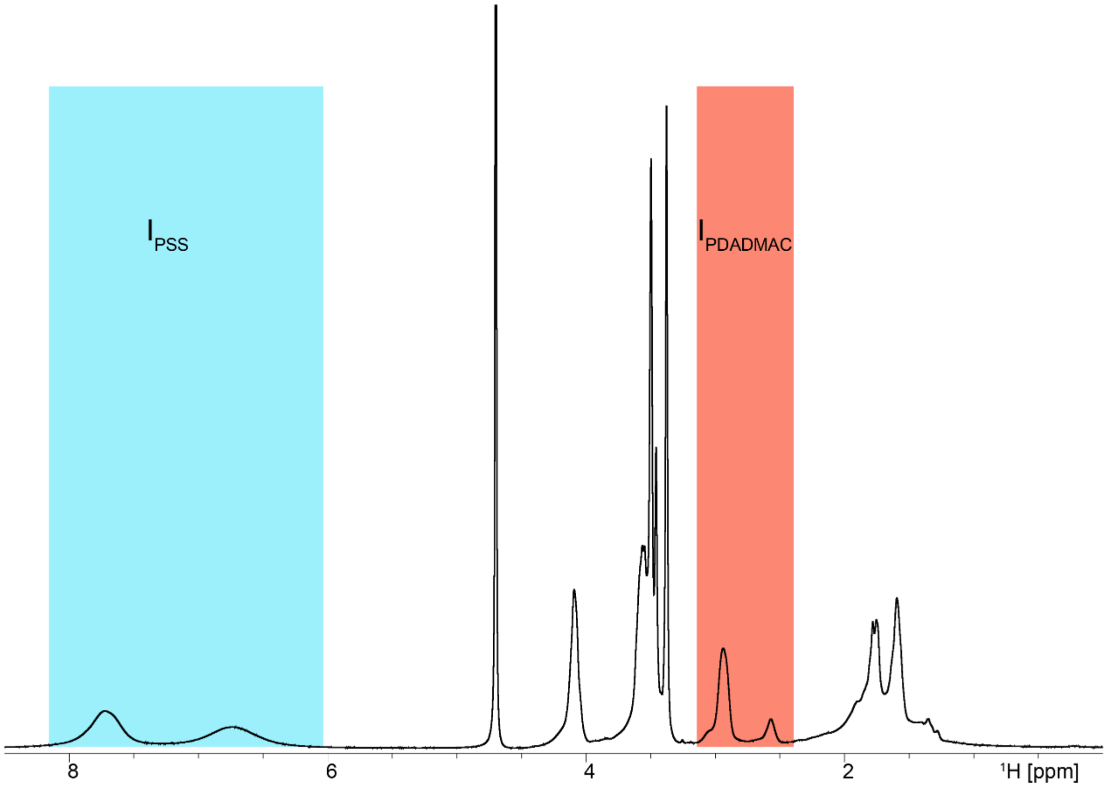

Figure S1*:* Demonstrative ^{1}H NMR spectrum of a PSS-PDADMAC membrane dissolved in 2.5 M KBr in D_2O. The regions in blue and orange correspond to the integration areas for PSS and PDADMAC, respectively.

2. Six compartment cell for resistance measurements

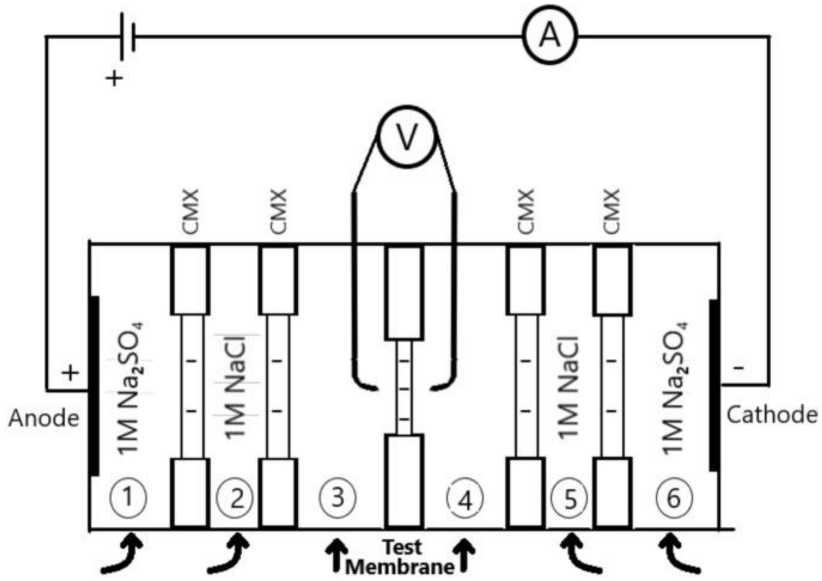

Figure S2: Scheme of the six-compartment cell used for resistance characterization. From Avci *et al.*[4]

3. Method for calculating fixed charge density

The fixed charge density of ion exchange membranes is defined as the molar charge per volume of the hydrated membrane. It is calculated using the ion exchange capacity (IEC) and the density of the hydrated membrane (ρ_m).

3.1. Ion Exchange Capacity (IEC):

The IEC was determined based on the composition of the saloplastic membranes, which was quantified using ^{1}H-NMR. The IEC is given by the following equation:

$$IEC\ (mol.g^{-1}) = \frac{w_{PDADMAC}}{MW_{PDADMAC}} - \frac{w_{PSS}}{MW_{PSS}} \qquad \text{Eq. S3}$$

Where:

- $w_{PDADMAC}$ and w_{PSS} are the the mass fractions of PDADMAC and PSS in the saloplastic membrane, respectively.
- $MW_{PDADMAC}$ = 126.2 g.mol^{-1} and MW_{PSS} = 183.2 g.mol^{-1} are the molecular weights of the PDADMAC and PSS monomers in the PEC (excluding counterions), respectively.

3.2. Hydrated Membrane Density (ρ_m):

The density of the hydrated membrane was calculated based on the known dry polymer density (1.27 g/mL)[5, 6] and the average water uptake (60%). The hydrated membrane density (ρ_m) was determined to be 1.17 g.mL^{-1}.

3.3. Fixed Charge Density:

The fixed charge density is then calculated as follows:

$$Fixed\ charge\ density\ (mol.L^{-1}) = IEC.\rho_m \qquad \text{Eq. S4}$$

Where:

- IEC is the ion exchange capacity (mol/g)
- ρ_m is the density of the hydrated membrane (1.17 g.mL^{-1}).

4. Elasticity of highly charged membranes

During permselectivity testing, the saloplastic membranes became slightly white and showed signs of stretching, as shown in **Figure S3**. This effect was more noticeable in membranes with higher PDADMAC content and when tested at higher electrolyte salt concentrations. The whitening of these transparent membranes indicates an increase in porosity. The thinning of the membranes, along with the formation of micropores or tears, explains the lower and unexpected trends in permselectivity

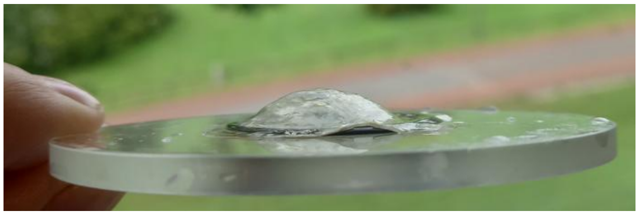

Figure S3: Photo depicting loss of mechanical stability of a saloplastic membrane prepared with 30 mol% excess PDADMAC after permselectivity measurement in 0.5 M KCl.

References

1. J. C. Yang, M. J. Jablonsky and J. W. Mays, *Polymer*, 2002, **43**, 5125-5132.
2. S. Donovan, A. J. Atkinson, N. Fischer, A. E. Taylor, J. Kieffer, J. P. Croue, P. Westerhoff and P. Herckes, *Environ Sci-Wat Res*, 2021, **7**, 1050-1059.
3. R. F. Shamoun, A. Reisch and J. B. Schlenoff, *Advanced Functional Materials*, 2012, **22**, 1923-1931.
4. A. H. Avci, C. Van Goethem, T. Rijnaarts, S. Santoro, M. Aquino, G. Di Profio, I. F. J. Vankelecom, W. M. De Vos, E. Fontananova and E. Curcio, *Molecules*, 2021, **26**.
5. H. M. Fares, Q. F. Wang, M. Yang and J. B. Schlenoff, *Macromolecules*, 2019, **52**, 610-619.
6. J. B. Schlenoff, M. Yang, Z. A. Digby and Q. F. Wang, *Macromolecules*, 2019, **52**, 9149-9159.
