# Supplementary figures and images for: Improving the fixed charge density of sustainably produced saloplastic anion exchange membranes
Source: RSC Sustain. 2025 Jun 17;3(8):3473–82. doi: 10.1039/d5su00221d (PMC12183635; doi:10.1039/d5su00221d)

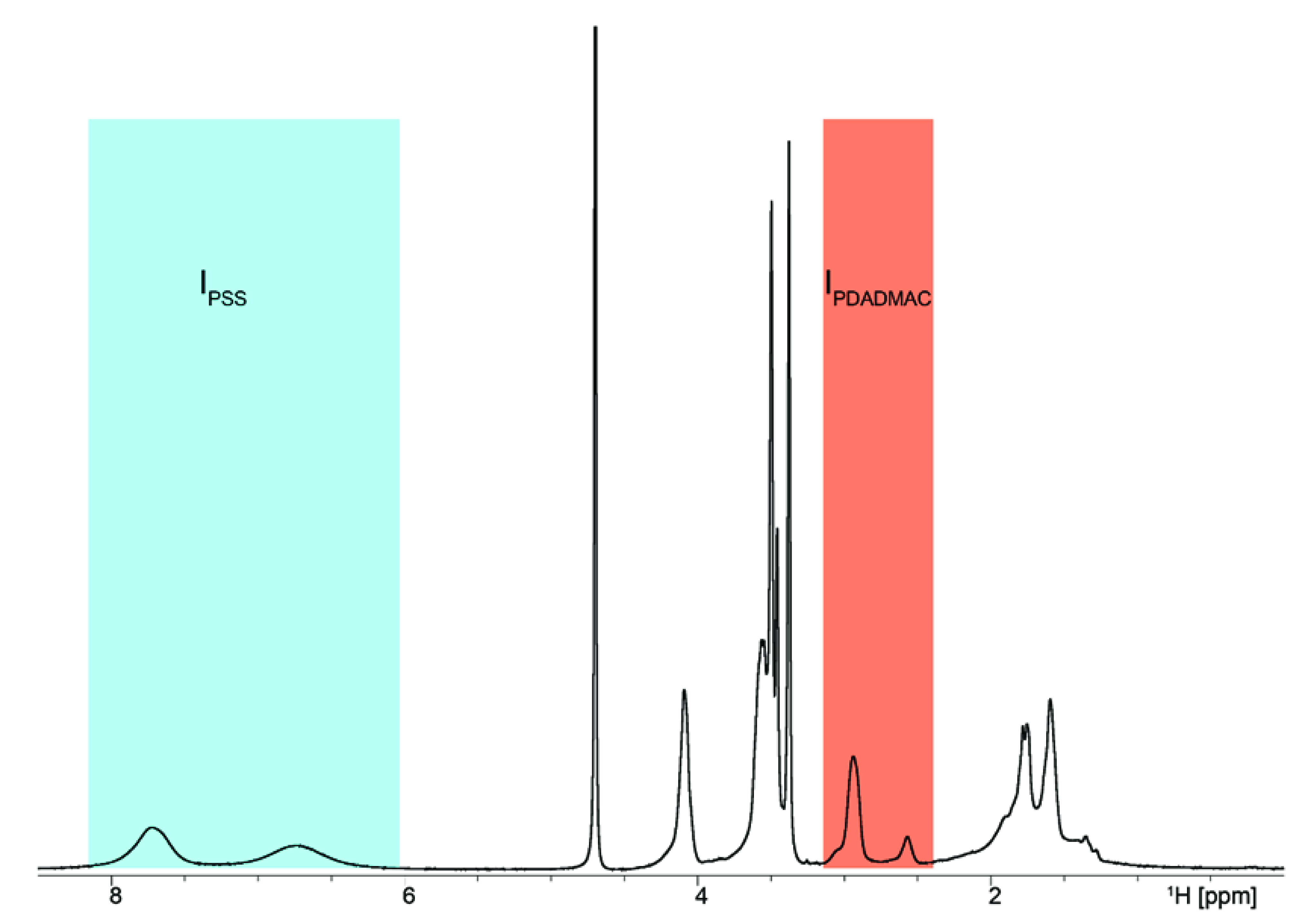

Supplement: SU-003-D5SU00221D-s001 [file SU-003-D5SU00221D-s001.zip › figure s1.tif]

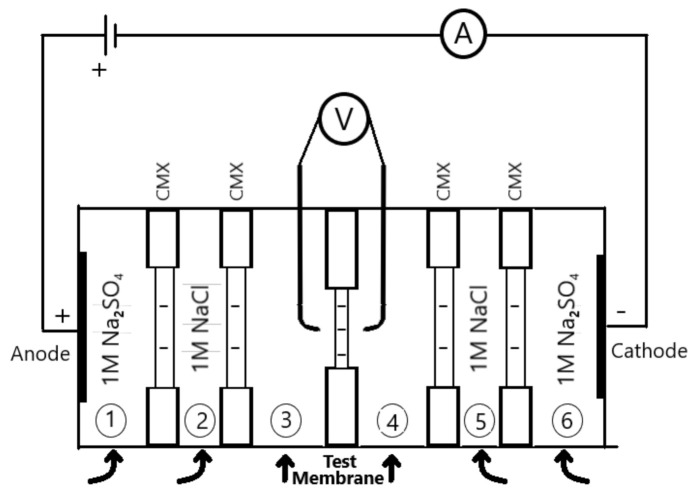

Supplement: SU-003-D5SU00221D-s001 [file SU-003-D5SU00221D-s001.zip › figure s2.tiff]

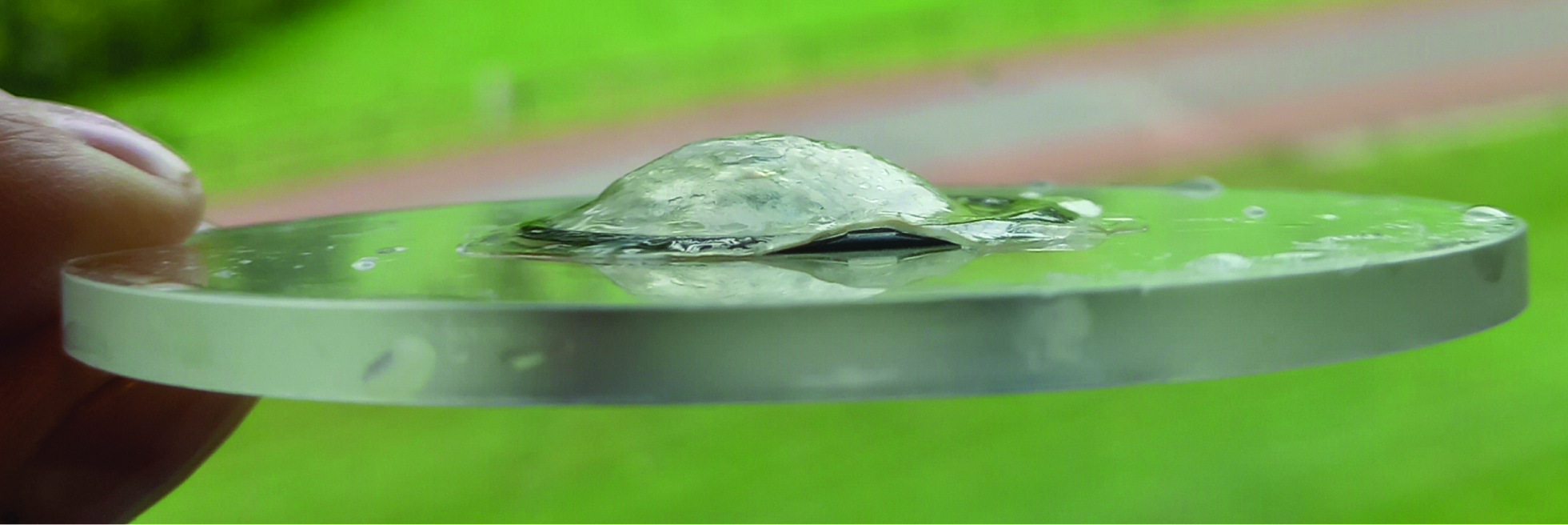

Supplement: SU-003-D5SU00221D-s001 [file SU-003-D5SU00221D-s001.zip › figure s3.tif]
